# Chronic typhoid infection and the risk of biliary tract cancer and stones in Shanghai, China

**DOI:** 10.1186/1750-9378-6-6

**Published:** 2011-05-02

**Authors:** Mahboobeh Safaeian, Yu-Tang Gao, Lori C Sakoda, Sabah M Quraishi, Asif Rashid, Bing-Shen Wang, Jinbo Chen, James Pruckler, Eric Mintz, Ann W Hsing

**Affiliations:** 1 Division of Cancer Epidemiology and Genetics, National Cancer Institute, Bethesda, Maryland, USA; 2 Department of Epidemiology, Shanghai Cancer Institute, Shanghai Cancer Institute, Shanghai, China; 3 Department of Epidemiology, University of Washington, Seattle, WA, USA; 4 Department of Pathology, the University of Texas MD Anderson Cancer Center, Houston, TX USA; 5 Department of Surgery, Zhongshan Hospital, Shanghai Medical University, Shanghai, China; 6 Department of Epidemiology and Biostatistics, University of Pennsylvania, Philadelphia, PA, USA; 7 Diarrheal Diseases Epidemiology Section, Foodborne and Diarrheal Diseases Branch, Centers for Disease Control and Prevention, Atlanta, GA, USA

## Abstract

Previous studies have shown a positive association between chronic typhoid carriage and biliary cancers. We compared serum *Salmonella enterica *serovar Typhi antibody titers between biliary tract cancer cases, biliary stone cases without evidence of cancer, and healthy subjects in a large population-based case-control study in Shanghai, China.

Participants included 627 newly diagnosed primary biliary tract cancer patients; 1,037 biliary stone cases (774 gallbladder and 263 bile-duct) and 959 healthy subjects without a history of cancer, randomly selected from the Shanghai Resident Registry.

Overall only 6/2,293 (0.26%) were Typhi positive. The prevalence of Typhi was 1/457 (0.22%), 4/977 (0.41%), and 1/859 (0.12%) among cancer cases, biliary-stone cases, and population controls, respectively.

We did not find an association between Typhi and biliary cancer in Shanghai, due to the very low prevalence of chronic carriers in this population.

The low seroprevalence of S. Typhi in Shanghai is unlikely to explain the high incidence of biliary cancers in this population.

## 

Biliary tract cancers, encompassing tumors of the gallbladder, extrahepatic bile ducts, and ampulla of Vater, are rare but highly fatal. Other than an association with a history of gallstones, the etiology of biliary tract cancer is obscure [1]. Several studies in India, a high-risk population for gallbladder cancer, have reported an association between chronic carriage of typhoid and biliary tract cancer [2,3]. Typhoid fever is caused by *Salmonella enterica *serovar Typhi, which can result in chronic typhoidal cholesystitis. About 3-4% of patients with acute Typhi infection become chronic, asymptomatic carriers [4]. To examine the relationship between chronic typhoid infection and biliary tract cancer, we compared serum antibodies to the Vi antigen of serovar Typhi, using the indirect hemagglutination (IHA) assay [5] between biliary tract cancer cases, biliary stone cases without evidence of cancer, and healthy subjects randomly selected from the general population, in a large population-based case-control study in Shanghai, China.

Details of the study have been reported elsewhere [6]. Briefly, between 1997 and 2001, using a rapid reporting system established in 42 collaborating hospitals in Shanghai, we enrolled 627 patients newly diagnosed with primary biliary tract cancer (ICD9 156), 1,037 biliary stone cases (774 gallbladder and 263 bile-duct) frequency-matched to cancer cases on age (5-year intervals), gender, and hospital, and 959 healthy subjects without a history of cancer, randomly selected from the Shanghai Resident Registry and frequency-matched to cancer cases on age (5-year intervals) and gender. Ninety-five percent of the eligible cases and 82% of the eligible controls participated in the study. The study protocol was approved by the Institutional Review Boards of the U.S. National Cancer Institute and the Shanghai Cancer Institute. All subjects provided written informed consent.

Determination of antibody status to the Vi capsular polysaccharide antigen of Typhi was assessed at the Centers for Disease Control and Prevention, Atlanta, GA. Titers ≥160 were defined as positive for chronic typhoid infection. For quality control, 30 samples were blindly retested; concordance of these samples was 100%.

We assessed the association of chronic typhoid infection with biliary tract cancer and stones by case-control status (i) overall, and (ii) by anatomic sub-site. Univariate and multivariate odds ratios and 95% confidence intervals were calculated, using maximum likelihood methods.

The characteristics of study subjects are shown in Table 1. Cancer cases (regardless of sub-site) were more likely to have gallstones compared with cancer-free controls. Table 2 shows the prevalence of chronic typhoid infection by subject status. Of the 2,293 subjects, only 6 (0.26%) were positive at ≥160 titer. Four additional participants (0.17%) were marginally positive (≥80 titer). The prevalence of Typhi was 1/457 (0.22%), 4/977 (0.41%), and 1/859 (0.12%) among cancer cases, biliary-stone cases, and population controls, respectively.

**Table 1:** Selected characteristics of cases and controls, Shanghai, China

			Biliary Tract Cancer						Biliary Stones			
			
Characteristics	Controls		Gallbladder		Extrahepatic Bile Duct		Ampulla of Vater		Gallbladder Stones		Bile Duct Stones	
						
	N	%	N	%	N	%	N	%	N	%	N	%
Total	859	100.0	262	100.0	140	100.0	55	100.0	728	100.0	249	100.0
Age												
<55	113	13.2	36	13.7	21	15.0	5	9.1	230	31.6*	56	22.5*
55 - 64	247	28.8	64	24.4	37	26.4	13	23.6	207	28.4	75	30.1
≥65	499	58.1	162	61.8	82	58.6	37	67.3	291	40.0	118	47.4
Sex												
Male	335	39.0	72	27.5*	84	60.0*	30	54.6*	250	34.3*	120	48.2
Female	524	61.0	190	72.5	56	40.0	25	45.5	478	65.7	129	51.8
Education (% > Middle school)	293	34.1	57	21.8	45	32.4	16	29.1	313	43.0	94	37.8
Smoking (%)†	259	30.2	69	26.4	63	45.0*	25	45.5*	177	24.3*	92	37.0
Alcohol Use (%)‡	177	20.6	42	16.0	45	32.1*	14	25.5	110	15.1*	48	19.4
Diabetes (%)	71	8.3	34	13.0*	12	8.6	3	5.5	78	10.7*	29	11.7*
Hypertension (%)	360	41.9	94	35.9	41	29.3*	14	25.5*	249	34.2	70	28.1*
Gallstones (%)	150	17.5	222	84.7*	93	66.4*	29	52.7*	-	-	-	-
Body Mass Index (% ≥25.0)	251	29.3	104	39.9	32	22.9	19	34.6	271	37.3	90	36.3

* Chi-sq test comparing cases and controls, p < 0.05

† Current smokers, defined as those who were smoking cigarettes regularly at the time of interview

‡ Current drinkers, defined as those drinking alcohol regularly at the time of interview

**Table 2:** Odds ratios* (ORs) and 95% confidence intervals (CI) for cancers of the biliary tract cancer and stones in relation to chronic typhoid infection, Shanghai China

	Biliary Tract Cancer					Biliary Stones			
		
	Controls^a^	Total	Gallbladder	Extrahepatic Bile Duct	Ampulla of Vater	Controls^b^	Biliary Stones	Gallbladder Stones	Bile Duct Stones
Typhoid	N	N OR (95%CI)	N OR (95%CI)	N OR (95%CI)	N OR (95%CI)	N	N OR (95%CI)	N OR (95%CI)	N OR (95%CI)
Total	859	457	262	140	55	654	977	728	249
									
Negative^c^	858	456	262	139	55	654	973	726	247
Positive									
≥1:160^d^	1	1 1.9 (0.1-31.1)	0	1 5.9 (0.4-95.2)	0	0	4	2	2

*OR = odds ratio, adjusted for age and for level of education

^a ^N = 800 for gallbladder cancer controls due to exclusion of those with history of cholecystectomy

^b ^Controls with gallstones were excluded.

^c ^Negative defined as no hemagglutination at <160.

^d ^Positive defined as hemagglutination at ≥ 160.

The low prevalence of chronic Typhi carriers in Shanghai largely explains the lack of association between biliary cancer and chronic carriage of Typhi in our study, suggesting that the rise in biliary tract cancer in Shanghai is likely due to other environmental or infectious agents. Biliary tract cancer is ranked 15^th ^in cancer incidence in China, which is similar to India (ranked 14^th^) and Bangladesh (ranked 12^th^). While it is hard to estimate incidence of typhoid fever, China is classified as a medium typhoid fever incidence country, compared with India which is classified as a high typhoid fever incidence country [7]. Thus, the very low prevalence of chronic typhoid carriage observed is consistent with the lower prevalence of acute typhoid fever reported in China compared with India and Pakistan [8], where high prevalence of Typhi chronic carriage has been reported (~2%). Misclassification of Typhi in cases and controls, due to the lower assay performance in a population where typhoid infection is rare (thus with lower titers among infected), is possible, although such misclassification should be non-differential among cases and controls. While the IHA assay has been well validated in high-prevalence chronic carriage settings, such as India, in a study from Vietnam, an area with high prevalence of acute typhoid fever, very low rates of serologic positivity among the general population were found [9], suggesting that the Vi-antibody test may be a less useful tool for detection of chronic carriage of Typhi in low-prevalence chronic carriage settings.

Our study is the largest population-based study of biliary tract cancer. With nearly complete case ascertainment and a high response rate, selection bias is minimal in the study. Misclassification of the outcome (cancer and stones) is also minimal, due to the stringent case confirmation.

In summary, due to the very low prevalence of chronic S. typhi carriers in this population, we were unable to evaluate chronic Typhi carriage and biliary cancer in Shanghai. In populations where chronic carriage of Typhi is rare, a much larger sample size and a more sensitive assay are needed to confirm the typhoid hypothesis. However, we are not able to rule out the possibility that chronic typhoid infection is associated with biliary tract cancer in populations with frequent outbreaks or a high prevalence of typhoid fever.

Given that gallstone is a common disease worldwide, affecting 15% of the population, and that over 17 million people worldwide are affected by typhoid fever, the role of Typhi infection in the development of gallstones and biliary tract cancer needs further clarification.

## Competing interests

The authors declare that they have no competing interests.

## Authors' contributions

MS: responsible for analysis and manuscript preparation. YTG: PI of the study (China), responsible for study design, subject recruitment, conduct of the study and all aspects of the study in China. BSW: surgeon, responsible for patient recruitments, diagnosis of cancer, and treatment of cases. EM and JP: - responsible for development of assays, QC of assays, running of assays and accuracy of results. AR: study pathologist. Responsible for the diagnosis of all cancer cases, QC, and review of slides. LS and SQ - responsible for the conduct of the study, data management, specimen management and retrieval, implementation of QCs. JC: contributed to the statistical analysis and design of the study. AH - responsible for all aspects of the study, including study design, subject recruitment, conduct of the study, analysis, and preparation of the manuscript All authors contributed to data interpretation and read and approved the final manuscript.
